# Cerebral Edema Leading to Subfalcine and Uncal Herniation in a Patient With Retinal Vasculopathy With Cerebral Leukoencephalopathy and Systemic Manifestations

**DOI:** 10.1177/19418744241310473

**Published:** 2024-12-20

**Authors:** Parker Hughes, Liang Lu, Michael Shi, Danial Syed

**Affiliations:** 1 Department of Neurology, 370833 Baylor College of Medicine, Houston, TX, USA; 2 Department of Medicine, 370833 Baylor College of Medicine, Houston, TX, USA

**Keywords:** cerebral edema, midline shift, retinal vasculopathy with cerebral leukoencephalopathy, corticosteroid, case report

## Abstract

Deterioration of a patient’s state of consciousness is among the most concerning signs encountered in clinical practice. The evaluation of this finding carries a broad initial differential diagnosis and must account for any relevant medical history. We describe the case of a 41-year-old male with known retinal vasculopathy with cerebral leukoencephalopathy and systemic manifestations (RVCL-S) who presented with progressive mental status decline and acute onset intractable headache. Head computed tomography (CT) revealed extensive vasogenic edema, resulting in right to left shift of 11 millimeters at the level of the lateral ventricles, with associated uncal and subfalcine herniation. He was treated with a 5-day course of methylprednisolone, leading to resolution of his lethargy and headache. Follow up neuroimaging with magnetic resonance (MRI) brain demonstrated interval improvement with the midline shift reduced to 3 millimeters after completion of high dose corticosteroids. Neurosurgical intervention was considered, but ultimately not required given his improvement. This case describes the management of life-threatening cerebral edema as a complication of RVCL-S disease progression. Due to the rarity of this disease, there are no standardized guidelines for treatment and the care for such patients relies on expert opinion, case studies, and extrapolation of principles learned from related conditions. Our intention is that the reporting of this case will contribute to the limited body of literature and aid those affected by this condition.

## Introduction

Retinal vasculopathy with cerebral leukoencephalopathy and systemic manifestations (RVCL-S) is a very rare genetic condition caused by a pathogenic variant of the *TREX1* gene. The disease is known for its widespread small-vessel disease affecting the retina, brain, liver, and kidneys. Cerebral vasogenic edema is a recognized manifestation of this disease process.^1^ Decline in the state of consciousness of a neurological patient with superimposed headache hints at the possibility of elevated intracranial pressure. Described here is an exceptional case of a patient with RVCL-S and cerebral edema causing uncal and subfalcine herniation with resolution of presenting symptoms after high dose steroids. Interval imaging also conferred improvement in the edema with treatment. A review of the pertinent literature on this topic is included.

## Case Description

Our institutional review board did not require approval for this case report though written consent was obtained from the patient.

**Figure 1.**
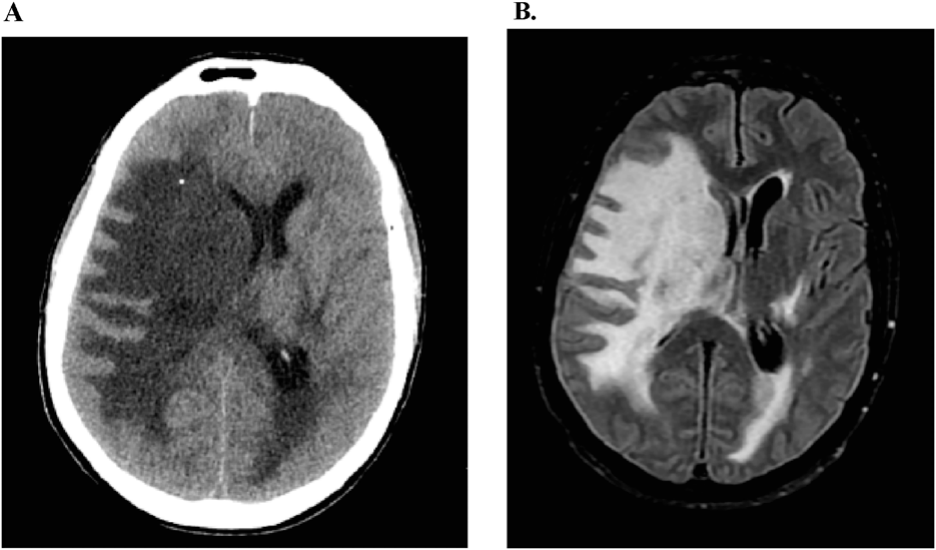
(A) Head CT at the time of presentation showing significant vasogenic edema burden at the subcortical white matter with associated 11 millimeter right to left midline shift at the level of the lateral ventricles. (B) MRI brain without contrast in T2 FLAIR sequence on day 2 of hospitalization after receiving 1 dose of methylprednisolone. Imaging was notable for extensive vasogenic edema bilaterally with the right side worse than left. There was an 8 millimeter right to left midline shift present and mild dilation of the left lateral ventricle concerning for entrapment.

A 41-year-old male with medical history notable for RVCL-S, chronic kidney disease, and hepatic hyperplasia presented to the emergency department for evaluation of headache and mental status decline. The patient had been assessed 2 days prior for headache and was given intravenous fluids, diphenhydramine, and prochlorperazine with some improvement. He was discharged home after this encounter. He has baseline deficits, including a left eye relative afferent pupillary defect, left eye visual acuity limited to counting fingers, left sided hemiparesis, and diffuse hyperreflexia. His mental status is normally alert and entirely oriented. On presentation, all chronic deficits remained apparent with the emergence of an acute lethargy demonstrating a Glasgow coma scale total score of 13 and component scores of E(2)V(5)M(6). Non-contrast head CT revealed extensive bilateral subcortical white matter hypoattenuation consistent with vasogenic edema driving right to left midline shift of 11 millimeters with subfalcine herniation (Figure 1(A)). There was also evidence of uncal herniation with effacement of the perimesencephalic cistern.

**Figure 2.**
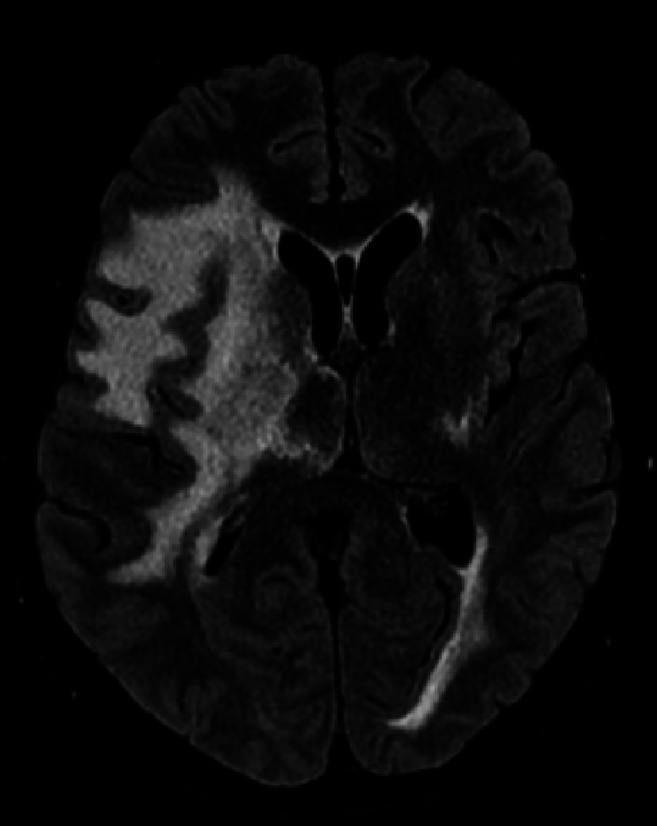
MRI brain without contrast in T2 FLAIR sequence on day 5 of hospitalization after completion of 5 dose methylprednisolone course. Imaging was remarkable for improvement of midline shift to just 3 millimeters.

Given this information, the patient was administered a dose of methylprednisolone 1000 mg due to the significant degree of cerebral edema. Neurosurgery was consulted for consideration of craniectomy, but this was deferred with election for close observation and a trial of medical management. The patient was admitted to the intensive care unit for hourly neurological checks. After the first dose of steroid, the patient reported subjective improvement in his headache and objectively his mental status returned to baseline. An MRI brain without contrast was completed on hospital day 2 showing extensive vasogenic edema in the bilateral cerebral hemispheres though more pronounced on the right side consistent with the earlier CT head. Mass effect of 8 millimeters midline shift and right uncal herniation were also present (Figure 1(B)). Gadolinium was not used because contrast imaging would not alter management as the RVCL-S diagnosis was already established and edema could be adequately assessed with FLAIR sequences. He was subsequently treated with methylprednisolone 1000 mg daily for an additional 4 days with resolution of headache by day 3 and continued stability in his neurological exam. A repeat MRI brain without contrast was obtained on day 5 after completion of the methylprednisolone course. This study revealed improvement in vasogenic edema and a reduction in the midline shift to only 3 millimeters (Figure 2).

The patient was discharged home on hospital day 6 with initiation of oral prednisone 60 mg daily with plan to taper down to 30 mg daily over the course of 4 weeks. Outpatient follow up to determine if additional down titration is appropriate remains pending. At last contact, the patient remained at his baseline level of function and has enjoyed being home while spending time with his spouse and children.

### Summary of the Existing Literature

We searched PubMed and Google Scholar for similar cases using search terms “retinal vasculopathy with cerebral leukoencephalopathy” AND “cerebral edema” OR “steroid” OR “glucocorticoid.” Of 18 abstracts screened, 11 articles were reviewed in full, and six were found appropriate for inclusion. Findings of these cases are outlined in Table 1.

**Table 1.** Summary of Literature Describing Management of Cerebral Edema in RVCL-S With Corticosteroids.

Author (year)	Age/Gender	Presenting Symptoms	Neuroimaging	Intervention	Outcomes
Stam et al^2^ (2016)	58 M	Right hemiparesis	MRI brain with ring enhancing lesions and left frontal lobe white matter edema	Dexamethasone 60 mg daily for 10 days	Minor improvement in hemiparesis
Jen et al^3,4^ (1997)	36 F	Blurred vision, headache, projectile vomiting	MRI brain with right hemispheric vasogenic edema and impending herniation	High dose dexamethasone for 3 days, then slowly tapered	Resolution of edema on imaging
Saito et al^5^ (2018)	30 M	Right hemiparesis	CT head with left frontal and temporal white matter edema	Corticosteroid of undisclosed type, dose, or duration	Resolution of hemiparesis and months-long period of stability
Sarkar, Way, & Verro^6^ (2012)	33 F	Worsening headache, blurred vision, facial weakness, left hemiparesis	Vasogenic edema with heterogenous lesions in bilateral hemispheres	Methylprednisolone	Near resolution of hemiparesis, dysarthria, and facial weakness
Muguruma et al^7^ (2022)	48 M	Aphasia, cognitive impairment, right hemiplegia	MRI brain with left parietal lobe edema and ring enhancement	Methylprednisolone 1000 mg daily for 3 days	Partial improvement in aphasia and right limbs attained anti-gravity
Raynowska et al^8^ (2018)	61 M	Gait disturbance, somnolence, hemiparesis	MRI brain with enhancing right frontal lesion, edema, and mass effect	High dose methylprednisolone	Improvement of hemiparesis

In brief, the literature surrounding management of cerebral edema in RVCL-S is quite limited, let alone the management of acute and potentially life-threatening edema. The cases included did not always indicate the therapy or its duration, but there appears to be consistency in a choice between use of methylprednisolone or dexamethasone with outcomes suggestive of an improvement in signs and/or symptoms. Reported cases provide a mixture of clinical and imaging outcomes.

## Discussion

RVCL-S is a rare genetic condition that affects the small vessels of highly vascularized tissues, especially those of the retina, brain, liver, and kidneys. This condition follows an autosomal dominant inheritance pattern with age-dependent penetrance due to pathogenic heterozygous C-terminal frameshift mutations in *TREX1* on chromosome 3p21.1-p21.3.^9,10^ Since 1988, a total of just 43 unrelated families have been identified with this disorder.^11^ The typical age of onset is between 35 and 50 years old with the most common presenting symptom of decreased visual acuity. Neurological signs may include paresis, cognitive changes, language deficits, visual field cuts, migraines, and seizures.^1^ Due to the systemic nature of this condition, patients will often also develop elevations of hepatic enzymes and chronic kidney disease. Other manifestations include, but are not limited to, hypertension, hypothyroidism, anemia, Raynaud phenomenon, and psychiatric disorders.^1^ Diagnostic criteria for RVCL-S has been proposed, though there are no consensus guidelines. Suspicion for the disease should be heightened as a possible unifier in those patients presenting with the constellation of demographics and symptoms as outlined previously. Abnormalities on an MRI brain limited to the white matter should be considered a feature consistent with RVCL-S.^2^ Unfortunately, no definitive treatment for the condition exists and care is limited to symptomatic management. Life expectancy is generally 5 to 20 years after onset of symptoms.^11^

There are ongoing clinical trials in the United States with the primary focus of better understanding disease progression and discovery of treatment options. Although outside the scope of this report, 1 trial is investigating a potential role with the P-selectin protein and possible intervention with crizanlizumab. There has also been consideration of gene editing with CRISPR, though this technique remains very much in its infancy. Previously examined therapies include cyclophosphamide and aclarubicin, though clinical trials were discontinued after no benefit was found^2,12^

Our patient at baseline carried many of the typical features of RVCL-S. Special concern was raised on presentation given his medical history and newly persistent headache with an altered state of consciousness. The finding of an 11 millimeter right to left midline shift on imaging driven by vasogenic edema provided an explanation for his symptoms. This is a recognized feature of disease progression.

The physicians involved in his care were presented with a management dilemma in the absence of evidence-based literature supporting specific treatment plans. Review of existing case studies suggested that a trial of high dose corticosteroids would offer the best approach. This was opted for in the form of methylprednisolone followed by prednisone taper. In the initial period, both mannitol and hypertonic saline were entertained as options using principles of cerebral edema treatment. This was ultimately not utilized due to a lack of evidence, though perhaps this could be an area of further study in the future. Hemicraniectomy was discussed as a potential intervention in the event of further deterioration of his neurological status, though there was fear that the bone flap may never be safely replaced. Fortunately, these measures were not needed as his presenting symptoms improved quickly with the initiation of steroid therapy. Literature review does not offer a precise mechanism for the role of steroids in acute management, though multiple case reports document efficacy. It would be reasonable to suspect the inflammatory component of RVCL-S would be tempered by steroid use.

This case highlights a life-threatening complication of cerebral edema in the setting of RVCL-S progression. Given the paucity of literature surrounding management of this scenario, it is our goal that future clinicians can use our experience to provide the best possible outcomes for the patients and families affected by this condition.
